# Novel nested conformal prediction analysis to unravel complexity in patient subtyping

**DOI:** 10.3389/frai.2026.1844254

**Published:** 2026-07-20

**Authors:** Linda Maldera, Marco Masseroli, Silvia Cascianelli

**Affiliations:** 1 Politecnico di Milano, Dipartimento di Elettronica, Informazione e Bioingengeria, Milan, Italy; 2 Computational Multi-Omics of Neurological Disorders (MIND) Lab Joint Research Platform, Fondazione IRCCS Istituto Neurologico Carlo Besta, Milan, Italy

**Keywords:** cancer, conformal prediction, machine learning, multi-label classification, patient subtyping

## Abstract

Patient subtyping is significantly challenged by intra-sample heterogeneity, which limits the effectiveness of traditional multi-class classification approaches enforcing mutually exclusive labels. Despite recent promising results in the transition from multi-class to multi-label classification, this process is not straightforward and proves hard to systemize, especially when working with datasets of small dimensions. Here, we design a novel approach, implemented in a computational framework, leveraging machine learning models and an original nested conformal prediction strategy for small datasets to enable a rigorous transition to multi-label classification. Conformal prediction can indeed offer a statistically sound approach for robust subtype predictions; yet, so far, it has been limited to be exploited only in large datasets, not often available in real biomedical scenarios. We evaluate our approach using breast cancer patient RNA-seq data from The Cancer Genome Atlas, with subtypes originally defined via the PAM50 classifier. From a methodological standpoint, our innovative nested strategy enables the successful application of conformal prediction, which commonly requires large datasets, to a relatively small sample size. Specifically, conformal prediction is adapted and applied both to the reference PAM50 classification and to multiple machine learning models to test coherence and compare the obtained multi-label predictions. Our results reveal subtype- and sample-specific complexity and highlight consistent patterns of ambiguity across models. Importantly, we distinguish between robust, inherently heterogeneous, and weak assignments, providing deeper insight into clear subtype confirmations and complexity, but also critical incoherent predictions. Thus, overall, this work demonstrates the utility of our framework for uncertainty-aware sample subtyping, particularly in small and highly unbalanced sample settings.

## 1 Introduction

Rapid advancements in computer science and AI, along with significant improvements in sequencing methods over the last few years, have enabled the increasing application of machine learning techniques in biology and medicine. As medicine shifts from a “one size fits all” paradigm to precision medicine, machine learning approaches on patient data could be a turning point for healthcare, improving our understanding of complex pathologies and supporting more personalized treatment strategies. In particular, patient subtyping based on molecular profiles has become a fundamental task for characterizing tumor biology and guiding clinical decision-making in oncology (Schlicker et al., 2012; Ringnér et al., 2016; Dai et al., 2015; Holm et al., 2017).

Despite these advances, significant challenges remain. A key limitation lies in the gap between predictive performance and clinical reliability, as high overall model accuracy does not necessarily imply the same level of correctness for any prediction at the individual patient level. This issue is further intensified by the limited sample size, high dimensionality of features and class imbalance of many omics datasets, which constrain the applicability of data-hungry predictive methods. Indeed, data availability still poses a great obstacle when large sample sizes are needed to train trustworthy machine learning models. In this context, quantifying and accounting for prediction uncertainty becomes as important as the prediction itself, especially when dealing with small datasets and clinically relevant decisions.

Furthermore, an additional and often overlooked challenge in cancer subtyping is the intrinsic heterogeneity of tumors (Ogino et al., 2012; Patel et al., 2014; Wang et al., 2017). Standard classification approaches typically adopt a single-label perspective, assigning each sample to a single subtype. However, growing evidence (Ma et al., 2018; Büttner et al., 2022; Marisa et al., 2021; Cascianelli et al., 2023) suggests that tumors frequently exhibit mixed molecular characteristics, reflecting intra-sample heterogeneity, clonal diversity, and microenvironmental influences. As a consequence, forcing single-label predictions may oversimplify the underlying biology and obscure relevant information. This motivates the need for approaches capable of supporting a transition from multi-class single-label to multi-label representations, where multiple subtype assignments can be simultaneously considered. The possibility of applying multi-label classification approaches for cancer subtyping is being explored in novel literature, particularly leveraging transcriptomic (Cascianelli et al., 2023, 2025; Zhang et al., 2025), and imaging (Salehzehi et al., 2025).

Conformal prediction (Angelopoulos and Bates, 2022) is a simple but effective method that highlights uncertainty in any sample prediction by generating prediction sets, which also enable the identification of samples that cannot be confidently assigned to a single subtype. In the last few years, conformal prediction has been used to address several clinical applications (Vazquez and Facelli, 2022), including subtyping tasks (Almaslukh, 2024; Pintawong et al., 2025) and estimation of diagnostic uncertainty (Olsson et al., 2022; Devetyarov et al., 2012) Conformal prediction expands the single-label output of a predictor with a set of possible labels, in which the presence of the reference class' label is guaranteed up to a certain confidence (usually 95% as α = 0.05). This can ease the transition from a simpler multi-class single-label classification to a multi-label setting, where the reference class' can be interpreted as the predominant, but often is not the only relevant one. Furthermore, beside facing prediction uncertainty, the size of the prediction set for a sample can also inform about the complexity of the sample classification. Thus, in the crucial context of cancer subtyping, conformal prediction can offer a parallel effort to support more exhaustive multi-label sample characterization and prediction uncertainty, strongly influenced by sample intrinsic heterogeneity, rather than simply due to model limitations. Accordingly, in this work, we have designed and implemented a computational framework made of two complementary phases and including a novel, systematic and nested usage of conformal prediction for a rigorous transition to multi-label classification in cancer subtyping. On one phase, conformal prediction analysis is repeatedly applied to the state-of-the-art classification method to evaluate prediction sets and categorize samples into robust, heterogeneous and weak based on their prediction patterns. On the other phase, an innovative approach is used to combine the training of multiple machine learning models with nested conformal prediction analysis. While classical conformal prediction would require high data availability to populate training, testing, calibration and validation sets, with a suggested number of at least 1,000 independent samples solely for the purpose of conformal threshold calibration (Angelopoulos and Bates, 2022), our novel nested strategy can overcome these limitations and enable working with a small and unbalanced dataset. Overall, the complementary phases of our framework lead to the comparison of multi-label classification results obtained across state-of-the-art and new machine learning models. But, more importantly, the framework allows for assessing reproducibility and statistical reliability, recognizing intrinsic sample heterogeneity from model-induced variability, shedding light on how model-dependent uncertainty interacts with data complexity. This perspective is especially relevant when training and testing machine learning on small-sample data, where uncertainty estimation is critical and traditional probabilistic outputs alone may be unreliable.

Our framework was applied to breast cancer subtype identification, where gene-expression-based classification of patients into specific molecular subtypes already supports clinical practice and tailored treatment (Sørlie et al., 2001; Parker et al., 2009; Wallden et al., 2015). Intrinsic breast cancer subtypes inferred using the state-of-the-art PAM50 method (Parker et al., 2009) were used as supervised information and therefore as reference labels' for conformal prediction analysis. PAM50 method considers the expression profiles of 50 target genes in a centroid-based algorithm to classify cancer patients in either Luminal A, Luminal B, HER2-enriched, Basal and possibly Normal-like subtypes. These intrinsic molecular subtypes have been found to have clear biological meaning and prognostic significance as discrete entities (Perou et al., 2000; Sørlie et al., 2001). Nonetheless, heterogeneous tumors benefit from being described using more than a single subtype and in general, any sample prediction may improve if provided with a clear indication of the uncertainty and complexity of the prediction itself.

## 2 Materials and methods

### 2.1 Dataset

The dataset used for our analysis was collected from the Cancer Genome Atlas (TCGA) (Weinstein et al., 2013) and comprises 1,053 samples of breast cancer RNA-seq expression profiles, with 25.150 genes per sample. The samples were assigned with *intrinsic molecular subtypes* using the state-of-the-art PAM50 Nearest Centroid Predictor (Parker et al., 2009), particularly its implementation described in Ciriello et al. (2015) and Cascianelli et al. (2020). These class assignments were also used as single-label references in our analyses, accounting for a total of 565 Luminal A, 157 Luminal B, 188 Basal, 72 HER2-enriched and 71 Normal-like samples. Luminal A tumors are typically estrogen receptor-positive, low-grade and associated with favorable prognosis, whereas Luminal B tumors also express hormone receptors but exhibit higher proliferation rates and generally poorer outcomes. HER2-enriched subtypes are characterized by over expression of HER2-related genes and are often more aggressive, although they can benefit from targeted therapies; however, they tend to be less frequent and harder to clearly distinguish. Basal-like tumors, often overlapping with triple-negative breast cancers, are highly proliferative and clinically aggressive. Finally, Normal-like subtypes, resembling non-tumor breast tissue profiles, remain biologically controversial in their interpretation (Sørlie et al., 2001; Prat and Perou, 2011; Ades et al., 2014; Perez, 2019).

### 2.2 Conformal prediction

Conformal prediction is a paradigm for creating statistically rigorous prediction sets in classification problems, valid in a distribution-free sense (Angelopoulos and Bates, 2022). It relies on a simple yet powerful mathematical approach that requires only a heuristic notion of uncertainty, derived from the model used to infer the class labels. To apply conformal prediction, it is necessary to define calibration and validation sets from data arising from the same distribution used for machine learning model training and testing. These are used to calculate a conformal score threshold, which, in turn, enables the definition of a prediction set of labels for each independent sample under analysis. To calculate the conformal scores, the selected classification model is applied to the calibration set, and a softmax function is used to transform the obtained prediction scores associated to all labels into a probability distribution as in Equation 1.


$$\begin{array}{l} c{\left(X\right)}_{j}=1-\frac{\exp X_{j}}{\sum_{i=1}^{k}\exp X_{i}} \end{array}$$ (1)

Where *c*(*X*)_*j*_ is the conformal score for subject *j* related to some class out of the *k* available classes, *X*_*j*_ is the models predicted value for that sample associated to the class and the denominator serves as a normalizing factor, summing over all class predictions for the same sample. The distribution of the conformal scores of the true class labels for all *n* samples in the calibration set is used to compute a threshold value, corresponding to the corrected (1−α) quantile of the distribution, as described in Equation 2.


$$\begin{array}{l} \hat{q}=\frac{\left\lceil \left(n+1\right)\left(1-\alpha \right)\right\rceil }{n} \end{array}$$ (2)

With the knowledge of the conformal score threshold, predictions on new data result in a multi-label ‘prediction sets' rather than single-label predictions, as all the class labels which satisfy Equation 3 will be considered as possible predictions. This guarantees the validity of the coverage property, which states that the probability of having the true class label in the prediction set 𝒞(*X*_*validation*_) is at least (1−α), as in Equation 4.


$$\begin{array}{l} C\left(X_{validation}\right)=\left\{y:s\left(X_{validation},y\right)\leq \hat{q}\right\} \end{array}$$ (3)

$$\begin{array}{l} P\left(Y_{validation}\in C\left(X_{validation}\right)\right)\geq 1-\alpha \end{array}$$ (4)

The size of the prediction set also gives valuable information regarding the reliability of the prediction itself, as predictions containing fewer elements can be considered “easier” and more reliable, while larger or null prediction sets can indicate complex or too uncertain predictions. The quality of the calibration can be assessed by analyzing the distribution of the prediction set sizes, which needs to be spread out between 0 and the maximum number of possible classes. This is due to the ability of the method to distinguish between easier and difficult predictions, but is also deeply influenced by the complexity of the tasks and heterogeneity of the samples. Thus, the correctness C of conformal predictions needs to be evaluated, defined as the empirical coverage for multiple runs on *R* different validation sets. This metric always needs to be distributed around (1−α) as described in Equations 5 and 6.


$$\begin{array}{l} C_{j}=\frac{1}{n_{validation}}\overset{n_{validation}}{\underset{i=1}{\sum }}1\left\{Y_{i,j}^{val}\in C_{j}\left(X_{i,j}^{val}\right)\right\}\text{for}j=1,...R \end{array}$$ (5)

$$\begin{array}{l} \bar{C}=\frac{\sum_{j=1}^{R}C_{j}}{R}\sim \left(1-\alpha \right) \end{array}$$ (6)

### 2.3 Feature selection, machine learning models, and performance metrics

Univariate feature selection with ANOVA (St and Wold, 1989) was performed for each analyzed training set, and 350 out of the 25.150 genes were used to train every model. The machine learning models used in our experimental settings were Logistic Regression (LR), Support Vector Machine (SVM), eXtreme Gradient Boosting (XGB), Random Forest (RF). To evaluate models' predictive performance, multiple metrics were used, such as accuracy, F1 score, Normalized Matthews Correlation Coefficient, Precision and Recall. Prediction sets obtained through conformal prediction analysis were further analyzed as multi-label predictions in comparison with PAM50-based multi-label prediction sets. To this aim, multi-label classification metrics were employed to evaluate the results of each combined model and conformal prediction strategy (Tsoumakas and Katakis, 2009; Mongardi et al., 2025), including multi-label accuracy, subset accuracy, multi-label sensitivity and specificity and relaxed accuracy (further details in Table 1).

**Table 1:** Multi-label metrics summary.

Metric	Formula	Meaning
Sensitivity	$\frac{1}{n}\overset{n}{\underset{i=1}{\sum }}\frac{\left|y_{i}\cap \hat{y_{i}}\right|}{\left|y_{i}\right|}$	Proportion of correctly predicted labels over all reference labels
Specificity	$\frac{1}{n}\overset{n}{\underset{i=1}{\sum }}\frac{\left|y_{i}\cap \hat{y_{i}}\right|}{\left|\hat{y_{i}}\right|}$	Proportion of correctly predicted labels over all predicted labels
Subset accuracy	$\frac{1}{n}\overset{n}{\underset{i=1}{\sum }}1\left[\hat{y_{i}}=y_{i}\right]$	Proportion of exactly predicted sets of labels
Multi-label accuracy	$\frac{1}{n}\overset{n}{\underset{i=1}{\sum }}\frac{\left|y_{i}\cap \hat{y_{i}}\right|}{\left|y_{i}\cup \hat{y_{i}}\right|}$	Proportion of correctly predicted labels over all predicted and reference labels
Relaxed accuracy	$\frac{1}{n}\overset{n}{\underset{i=1}{\sum }}\left[y_{i_{primary}}\subseteq \hat{y_{i}}\right]$	Proportion of presence of the primary label in the multi-label set

For each of the *n* available samples with single-label assignment *y*_*primary*_, the multiclass ground truth *y*, and the multiclass prediction $\hat{y_{i}}$ consist of a set of labels, among all the *q* possible labels.

## 3 Computational framework

**Figure 1:**
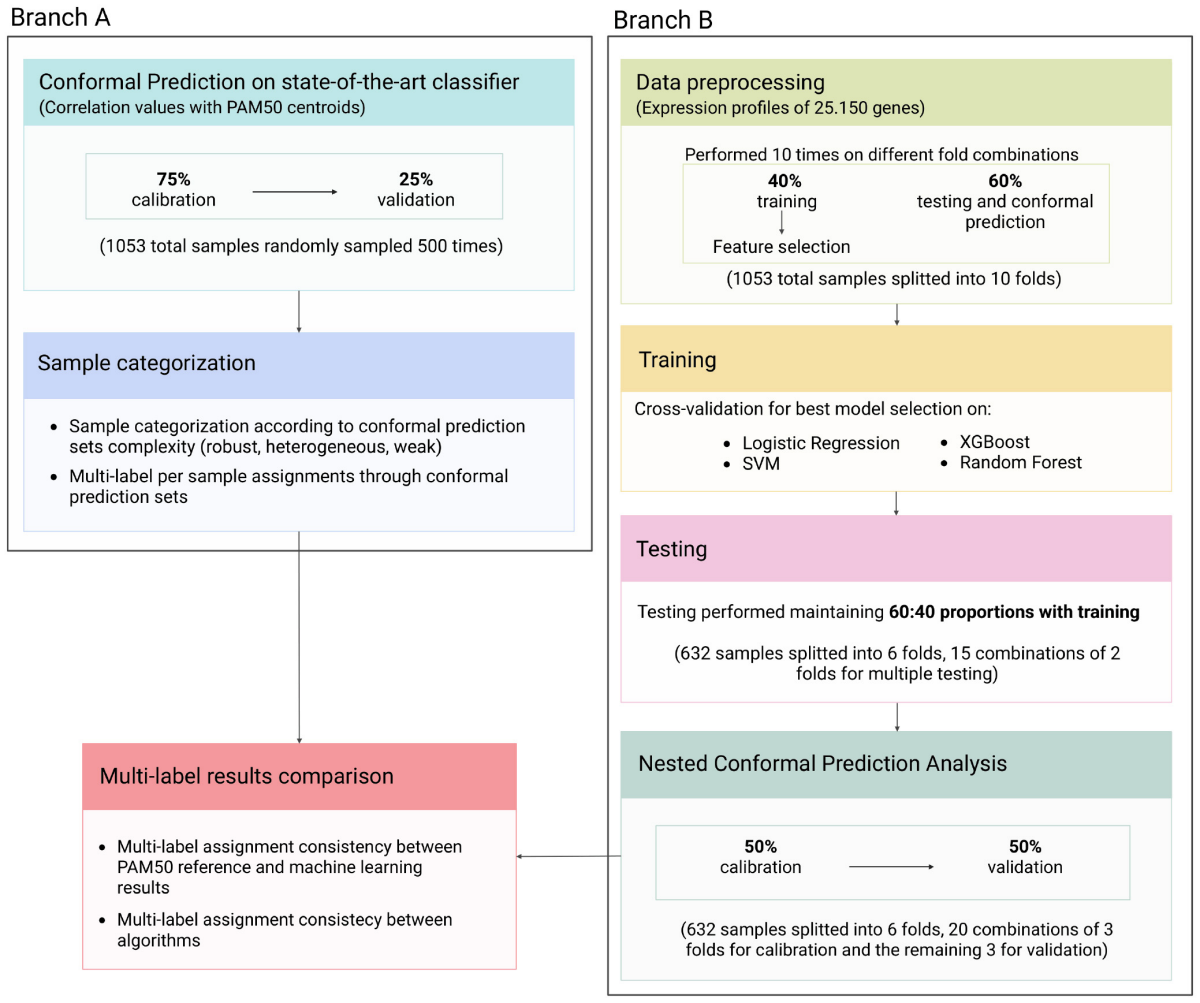
Computational framework. Nested conformal prediction strategy (NCPA). **(A)** Illustrates the application of conformal prediction on the state-of-the-art classification method, which consists in multiple random splits of the original dataset with a 75:25 proportion for calibration and validation, allowing to assign multiple prediction sets to all samples, and performing sample categorization into either robust, heterogeneous or weak categories. **(B)** Illustrates the application of our Nested Conformal Prediction Analysis with multiple machine learning algorithm types. The workflow highlights data preprocessing, training with cross-validation, testing with appropriate proportions with respect to the training set and the final application of conformal prediction calibration and validation. Prediction sets are then compared as multi-label assignments in the two branches.

This section outlines the computational framework of our study, which includes two complementary analytical branches, as reported in the overview diagram provided in Figure 1. The main methodological contribution lies in our systematic and nested strategy of conformal prediction: this was designed to work with a relatively limited amount of samples while offering a rigorous transition to multi-label classification in cancer subtyping.

In the branch A, to assess the adequacy or limitation of each assigned single-label reference, we analyze directly the results of the state-of-the-art classifier, i.e., the values of similarity of each sample to the five PAM50 centroids in our use case. These values were converted into softmax scores. In this workflow, we consider an already trained state-of-the-art classifier (here the PAM50 with its known centroids), and the entire dataset can therefore be devoted to conformal prediction analysis (CPA) only. To avoid biases that could occur in the calculation of the conformal threshold due to the splitting of the dataset in calibration and validation, this splitting is randomly performed 500 times, with 75% of the samples used for calibration and the remaining 25% for validation. The proportion 3:1 for the two sets was chosen after a preliminary evaluation of the variability that could arise in the conformal threshold with smaller calibration sets. The results of the prediction sets obtained after CPA, allow us to divide the samples into three categories of interest, i.e., robust, heterogeneous, or weak predictions, according to the rate at which they are correctly classified. When the single-label reference, here inferred by the PAM50, is the only element in the prediction sets with a frequency equal to or above 90%, the samples are categorized as robust. When the prediction sets include multiple subtypes while the single-label reference appears alone in less than 90% of the cases, but more then 10% the samples are categorized as heterogeneous. Lastly, when the single-label reference appears alone in the prediction set with a frequency below 10%, the samples are considered weak. Besides this sample prediction categorization, all the samples are also associated with their multi-label reference characterization derived from the prediction sets obtained after CPA. Robust samples maintain their single-label; heterogeneous samples are associated with their found broader prediction set; while weak samples are assigned with no label at all, to recognize them in further evaluations.

In parallel, in the branch B of the framework, CPA is used on machine learning models trained to perform the same single-label subtyping task of the PAM50 method. To correctly perform conformal prediction, a dataset needs to be partitioned in training, testing, calibration and validation sets; these partitions are used for conformal threshold calculation and generation of the prediction sets, respectively. In order to sample the entire dataset and overcome the issues of having only few samples available in splitting the dataset four times, we have designed an analytical workflow of multiple stratified sampling and splittings, followed by what we named Nested Conformal Prediction Analysis (NCPA). This strategy allowed us to obtain multi-label predictions for each sample of our dataset, alternating multiple independent data splitting and analyses. All the details are reported in the next subsection. Lastly, all the multi-label assignments obtained from machine learning predictors are tested against the ones obtained from the state-of-the-art method in branch A of the framework and against each other, in order to establish to what extent the sample predictions and categories remained consistent.

### 3.1 Analytical strategy with novel nested conformal prediction

**Figure 2:**
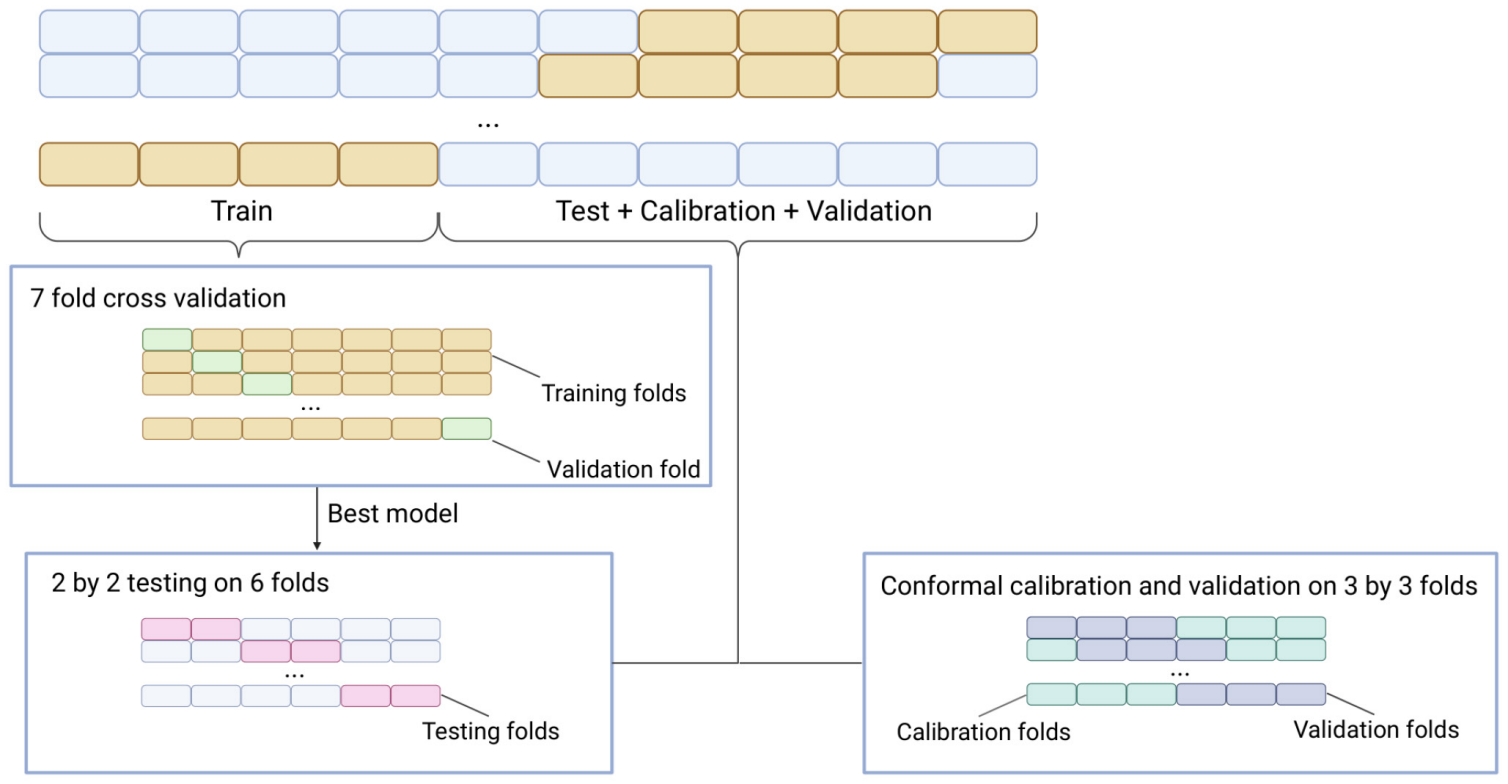
Data splitting for stratified machine learning pipeline. Representation of the created structure for the dataset. The dataset is splitted in 10 primary folds, allowing the minority class to be correctly represented in each fold; 40% of the dataset (421 samples) is used for finding the best machine learning model for each model type, with a 7-fold nested cross validation. The remaining 60% of the dataset (632 samples) is used for testing, to maintain a proper balance between training and testing sets (60:40), testing is performed exhaustively on 15 combinations of two 6-folds. This same proportion of the dataset is also used for conformal prediction, by exploiting the 20 possible combinations of three 6-folds. Therefore, 50% of the testing dataset (316 samples) is used for calibration, calculating the conformal prediction threshold, and the remaining 50% is used for validation, thus creating the multi-label assignment through the calculation of prediction sets. This nested structure allows for all the 1,053 samples of the dataset to have multiple multi-label assignments.

Our analytical strategy requires multiple stratified splits and sampling of the original dataset, combined with a novel nested conformal prediction analysis, as synthesized in Figure 2.

- For N run (*N* = 10 by default), the dataset is splitted in 10 stratified folds, named “primary folds.” The number of folds is decided allowing adequate representation of the minority class through all steps of the pipeline. For each run, a training set is built by a random combination of four primary folds, while the remaining six primary folds are left aside for testing and NCPA.
- To find both the best hyperparameters and parametrization for each model, training is performed on each training set using k-fold cross-validation, with k such that each fold contains around 5% of the initial dataset size. In this way, the best performing model is obtained for each model type.
- The remaining six primary folds are first used for testing the obtained best models. Specifically, 15 testing sets are used exploring all the combinations of two six primary folds (unordered and without repetition) exhaustively. This enables evenly sampling all the data not used for training and compute average metrics and standard deviations on testing metrics, while maintaining a 60:40 proportion of training and testing sets.
- The same six primary folds are also used for our nested calibration and validation of conformal thresholds during the NCPA. Specifically, all the 20 three combinations of the 6 primary folds are used once for calibration and once for validation. Applying this NCPA methodology allows for obtaining a distribution of 20 different conformal thresholds for each model and run, which are applied to their corresponding validation set, defining a distribution of prediction sets for each sample.

Thus, after performing NCPA, each of the 1,053 samples in the dataset results in having numerous prediction sets. For each sample, the most common prediction set is ultimately considered as the multi-label classification of the corresponding model. In case of tied results, the prediction set with the most number of labels, i.e., the most complex one, is chosen.

## 4 Results

### 4.1 Conformal prediction on PAM50 and multi-label PAM50 prediction sets

The used PAM50 single-label references were obtained as the maximum of all the correlation values with respect to the five known subtype centroids (one for each molecular intrinsic subtype Parker et al., 2009). The data in this analytical branch were divided only into calibration and validation sets, testing 500 different random splits. In fact, no training phase was needed for PAM50 and conformal prediction analysis was used directly on the conformal scores obtained, as described in Equation 1, from the correlation values, where the reference label, a.k.a. the reference class', always corresponds to that with the maximum correlation value and softmax score. Across the 500 runs, the conformal thresholds remained highly stable, with an average value of 0.7510 ± 0.0009.

The prediction sets obtained across all validation sets were then aggregated to derive a PAM50-based multi-label characterization for each sample in the dataset. Each sample was assigned the prediction set with the highest appearing frequency. In this way, each sample was no longer described exclusively by its original single-label PAM50 subtype, but possibly by the all the subtype labels consistently found with our exhaustive CPA.

**Figure 3:**
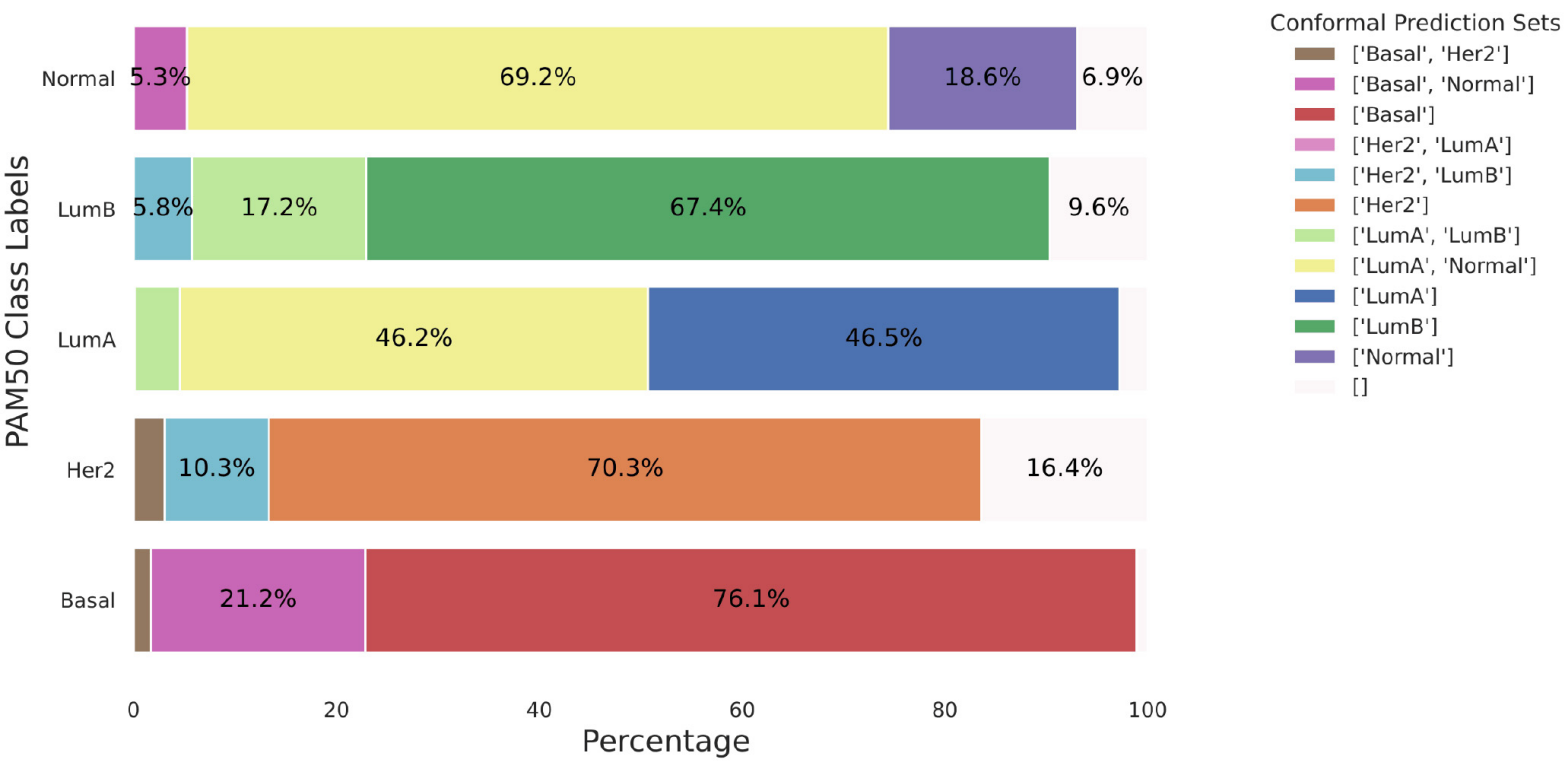
PAM50 prediction sets distribution with respect to ground truth labels.

These multi-label prediction sets, depicted in Figure 3, were compared to the reference PAM50 single-label classification of the dataset, as detailed in Supplementary Table S1, and summarized in Table 2. Particularly, Table 2 reports the categorization of samples into *robust, heterogeneous*, and *weak* groups in comparison to their original PAM50 subtype assignment.

**Table 2:** PAM50 based single-label vs. multi-label dataset composition.

PAM50 single label	Count	Robust	Heterogeneous	Weak
LumA	565 (53.66%)	249 (44.07%)	298 (52.74%)	18 (3.19%)
Basal	188 (17.85%)	145 (77.13%)	40 (21.28%)	3 (1.60%)
LumB	157 (14.91%)	101 (64.33%)	42 (26.75%)	14 (8.92%)
HER2	72 (6.84%)	47 (65.28%)	10 (13.89%)	15 (20.83%)
like	71 (6.74%)	12 (16.90%)	54 (76.06%)	5 (7.04%)

PAM50 state-of-the-art single-label classification (on the left) compared to sample categorization into robust, heterogeneous and weak samples based on multi-label prediction set obtained over 500 repetitions of conformal prediction analysis (on the right).

PAM50 Basal tumors showed the highest proportion of robust samples (77.13%), consistent with the fact that this subtype is generally considered one of the most transcriptionally distinct and therefore more readily identifiable in molecular classification settings (Haupt et al., 2010). In contrast, PAM50 Luminal B tumors displayed a substantial proportion of heterogeneous cases (26.75%), most frequently mapping to mixed LumB/LumA prediction sets. A comparable absolute number of such heterogeneous LumA/LumB cases was observed for PAM50 Luminal A tumors; however, because PAM50 Luminal A represents the largest fraction of the dataset (53.66%), the relative proportion of heterogeneous LumA/LumB assignments within the PAM50 Luminal A class remained much lower, around 5%. Overall, this suggests the presence of a biologically plausible sample inner heterogeneity between the two luminal classes, with ambiguous cases from both directions not reflecting a preferential shift from one subtype to the other (Ades et al., 2014; Perez, 2019). PAM50 HER2-enriched tumors, instead showed the highest percentage of weak samples (20.83%), pointing out a greater difficulty in obtaining a stable and confident characterization for this class within the multi-label context. While the absolute number of weak PAM50 HER2-enriched samples was similar to that observed in PAM50 Luminal A and Luminal B, their relative proportion was markedly higher, given the limited number of PAM50 HER2-enriched samples. This pattern reflects the higher uncertainty in recognizing HER2-enriched traits and, overall, suggests that the amount of weak samples does not seem to be dependent on class dimensions, but rather on the classification intricacy. PAM50 Normal-like class exhibited the lowest proportion of robust samples (16.90%), while a striking 76.06% of cases fell into the heterogeneous group. Most of these heterogeneous samples (over 70%) were assigned to the Normal-like/Luminal A prediction set, while a smaller but still notable fraction (7.04%) was associated with the Normal-like/Basal combination, which is very biologically distant from the Normal-like/Luminal A axes. These findings highlight the difficulty of characterizing the Normal-like subtype as a clearly distinct entity and reflect the long-standing uncertainty surrounding its biological and clinical interpretation. Indeed, the Normal-like class has often been regarded as controversial, as it may in part capture normal tissue contamination or non-tumor-like transcriptional signals rather than a fully distinct tumor subtype. Its strong overlap with Luminal A, the subtype generally associated with the most favorable prognosis, is therefore particularly notable and was worthy of further investigations in the branch B of our analytical framework. In fact, this mixed behavior in multi-label characterization also heavily affected the PAM50 Luminal A class itself, reducing its proportion of robust samples. More than 52% of Luminal A cases were classified as heterogeneous, with 47.43% of prediction sets composed by Normal-like and LumA labels together. This provides further evidence that the Normal-like label should be interpreted with caution and motivates a more detailed investigation of these classes and mixed patterns in the analyses of our branch B focused on machine learning based classification models. In the following, the results of these machine learning models will be discussed using as references alternatively, the reference class' reference given by the PAM50 single-label classification (also used as supervised information for the training phase) and the new multi-label characterizations derived from the PAM50-based prediction sets returned by our exhaustive conformal analysis in branch A.

### 4.2 Machine learning results

As summarized in Figure 2 training was repeated 10 times on different random samplings, each time accounting using a stratified 40% of the entire dataset. Within each of the 10 independent machine learning runs, feature selection was performed separately using ANOVA, and the resulting feature space including 350 genes was used by all model types trained within the same run. Due to the variability of feature selection in small training datasets, a total of 582 unique genes were found by combining the features selected across all runs: out of these 582 genes, 192 were selected in all the 10 runs, while 288 belong to more than the 70% of the runs. On each training set, all models were trained to perform the original single-label subtyping task, i.e., of recognizing the PAM50 reference label. This ensured that the subsequent nested conformal prediction analysis (NCPA) and the extraction of multi-label prediction sets remained fully independent from the conformal analysis previously applied to the PAM50 classification. Models are tuned and trained using 7-fold stratified cross-validation to obtain a final predictor for each family of models. Testing was then carried out on multiple stratified sets derived from the remaining part of the dataset, partitioned into 6 folds, with 2 folds at a time used as a single test set. For each trained model, this procedure ensured 15 distinct test sets, each corresponding to approximately 20% of the full dataset, enabling the estimation of performance distributions rather than single-point results. Moreover, since the entire training/test splitting procedure was repeated in 10 independent runs, the mean and standard deviation of all the collected performance metrics also reflect this additional source of variability, providing robust and unbiased estimates. The results obtained for all models are summarized in Table 3. Logistic regression achieved the best overall performance for the single-label classification task, with the highest values of accuracy, normalized Matthews correlation coefficient (MCC), recall, and F1 score.

**Table 3:** Machine learning evaluation metrics in single label subtyping.

Model	Accuracy	MCC	Precision	Recall	F1 score
LR	0.881 ± 0.02	0.816 ± 0.032	0.895 ± 0.019	0.881 ± 0.02	0.886 ± 0.019
RF	0.854 ± 0.016	0.77 ± 0.026	0.904 ± 0.014	0.854 ± 0.016	0.869 ± 0.014
SVM	0.868 ± 0.019	0.796 ± 0.029	0.885 ± 0.02	0.868 ± 0.019	0.872 ± 0.018
XGB	0.861 ± 0.018	0.781 ± 0.028	0.899 ± 0.016	0.861 ± 0.018	0.873 ± 0.016

Overview of the 10 machine learning runs, calculated on the 15 combinations of 2 out of 6 folds. Best SVM model was defined in each of the 10 runs, resulting in a polynomial kernel on seven runs and a linear one on the remaining 3.

#### 4.2.1 Nested conformal prediction analysis and comparison of multi-label prediction sets

**Figure 4:**
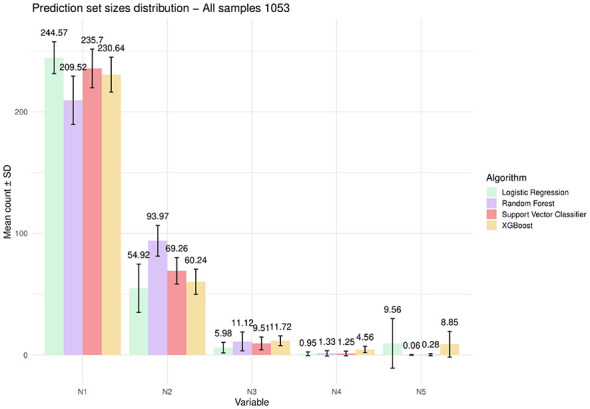
Prediction set size distribution for all samples. Mean and standard deviation of number of prediction sets sizes for all samples and all runs of NCPA for all tested algorithms. The majority of prediction sets consist of only 1 or 2 labels, while prediction sets with more than three labels are found only in few samples.

Nested conformal prediction was successfully applied to the best machine learning models of every run, yielding mean coverages consistent with the theoretical expectation of 95% for all model types (logistic regression 95.71 ± 1.42, XGBoost 95.59 ± 1.40, SVM 95.64 ± 1.48, and random forest 95.51 ± 1.49). The distribution of prediction set sizes across algorithms is shown in Figure 4. Logistic regression shows the highest proportion of single-label prediction sets. consistent with its strong classification performance. However, it also showed greater variability in prediction set sizes, as reflected by the higher standard deviation in the number of prediction sets composed by two or even all five labels. This suggests that logistic regression may be more sensitive to small variations of conformal thresholds arising in specific data splits. In particular, for relatively permissive thresholds set under specific calibration cases, the resulting class scores of this linear model may sometimes produce less pronounced separation between the dominant class and the other classes, thereby allowing many labels to be retained in the conformal prediction. Yet, prediction sets containing four or five labels can be interpreted as indicating particularly problematic or non-informative predictions, a phenomenon conceptually comparable to the absence of confident assignments observed in the weak samples obtained in the PAM50-based conformal analysis.

Nonetheless, prediction set with three or more labels constitute a clear minority across all the models. In particular, prediction sets composed of four or five labels reflect marked weakness in the prediction and may therefore be considered conceptually comparable to the weak samples with no-assignments identified in the PAM50-based conformal analysis. This hypothesis was confirmed by analyzing the frequency of prediction sets with three or more labels in each of the three characterization classes. For heterogeneous and robust samples, the frequency of such prediction sets is respectively (2.51 ± 4.01)% and (4.41 ± 3.86)%, while for weak samples this percentage rises to (33.59 ± 19.67)%. NCPA results indicate both the generally good performance of the underlying single-label classifiers and the relevance of adopting a multi-label perspective. In particular, while highly ambiguous predictions were infrequent, a meaningful proportion of samples was still consistently associated with more than one plausible subtype, supporting the need for a multi-label classification. Furthermore, the prediction set size distributions were overall quite coherent across algorithms and consistent across runs, supporting both the intrinsic value of the found results and the robustness of the here proposed analytical framework. Prediction sets were also tested against the multi-label assignments previously obtained with the independent application of CPA on the PAM50 classification. Notice that, in this comparison, PAM50-based weak samples were excluded from the evaluation given their null reference assignment, but studied separately to assess the predictive behavior of the machine learning models in this sample group.

### 4.3 Heterogeneous and robust samples

In this subsection, the multi-label evaluation is restricted to *robust* and *heterogeneous* samples as defined by the CPA performed directly on PAM50, while weak samples were discussed in the next subsection. Multi-label metrics calculated on heterogeneous and robust samples (as defined from conformal analysis on PAM50) are summarized in Supplementary Figure S1. Interestingly, all metrics showed only limited fluctuation across runs, as reflected by the small standard deviations obtained despite the multiple layers of variability introduced by NCPA and repeated resampling. This overall stability further supports the robustness of our proposed framework. Relaxed accuracy represents the frequency at which the original PAM50 single label is included in the prediction set and therefore corresponds to the notion of coverage in conformal prediction. Since the significance level used to compute the conformal threshold was set to α = 0.05, and the subset of weak, more complex, samples was excluded from the present analysis, the relaxed accuracy reached peaks of 0.96–0.97, slightly above the nominal 0.95 expected if all samples had been retained.

Once again, logistic regression appears to be the best-performing algorithm overall, mainly for its higher specificity and subset accuracy, despite the other models also reach comparable performance metrics overall. Subset accuracy of logistic regression reached a value of 0.58 ± 0.02 that, though may appear low, reflects the ability of the model to predict the *exact* multi-label assignment and therefore represents a highly stringent criterion. These values, almost comparable also for the other model types, suggest the presence of non-negligible discrepancies between the PAM50-based multi-label characterization and the prediction sets generated by the different machine learning models. However, subset accuracy must be evaluated together with multi-label accuracy, which was around 0.75 across all algorithms, and captures also partial overlap between predicted and reference label sets rather than requiring an exact match. In this context, a multi-label accuracy of 75% can be considered substantial, especially given the fully independent and exploratory nature of the conformal prediction analysis applied to derive the PAM50- multi-label characterizations. Thus, despite exact reproduction of PAM50-based multi-label characterizations being limited, the classifiers are still able to capture a large fraction of the underlying subtype complexity. In addition, specificity was consistently higher than sensitivity across all the models, indicating that the classifiers tend to generate prediction sets that are, on average, smaller than the corresponding PAM50-derived multi-label sets. This appears as a relatively more conservative predictive behavior, where models tend to retain only the most supported labels rather than expanding prediction sets. From a methodological perspective, this may be advantageous, as it suggests that prediction sets of machine learning models are less prone to overestimating complexity when the evidence for multi-label assignments is not considered strong enough.

Robust samples were further analyzed separately to investigate the behavior of each tumor class in cases where the PAM50-based conformal analysis suggested a quite stable single subtype characterization. For these samples, the number of exact, incorrect, and partial matches was counted, together with the frequency of complex prediction sets containing three or more labels. The results of this analysis are summarized in Supplementary Table S2. As expected, Basal tumors showed the highest proportion of exact predictions, followed by Luminal A. More generally, for these robust samples, the percentage of completely incorrect predictions remained low across all algorithms, supporting the validity of the PAM50-based conformal categorization for these cases. HER2-enriched tumors showed a less stable behavior across classifiers. In particular, XGBoost and Random Forest more frequently assigned complex prediction sets to HER2-enriched samples, whereas logistic regression performed more consistently also in this class. This observation is coherent with the greater difficulty already noted in clearly delimiting HER2-enriched tumors (Cascianelli et al., 2020, 2025), whose molecular profile may overlap with neighboring subtypes. The 12 Normal-like samples belonging to the robust category showed high proportions of both complex and incorrect predictions, further highlighting the difficulty of distinguishing this class, already emerged from the conformal analysis on PAM50, where the Normal-like class already displayed a very low proportion of robust assignments and a large number of heterogeneous cases (especially involving the mixed Normal-like/Luminal A and Normal-like/Basal prediction sets). Taken together, these findings suggest that the uncertainty associated with the Normal-like class may not be fully explained by intrinsic biological heterogeneity alone, but may instead reflect the controversial nature of this subtype, considered by the PAM50 method, but discarded in other proposed classification systems (Wallden et al., 2015; Perez, 2019). This interpretation is further supported by the dedicated analysis of heterogeneous samples described below.

**Figure 5:**
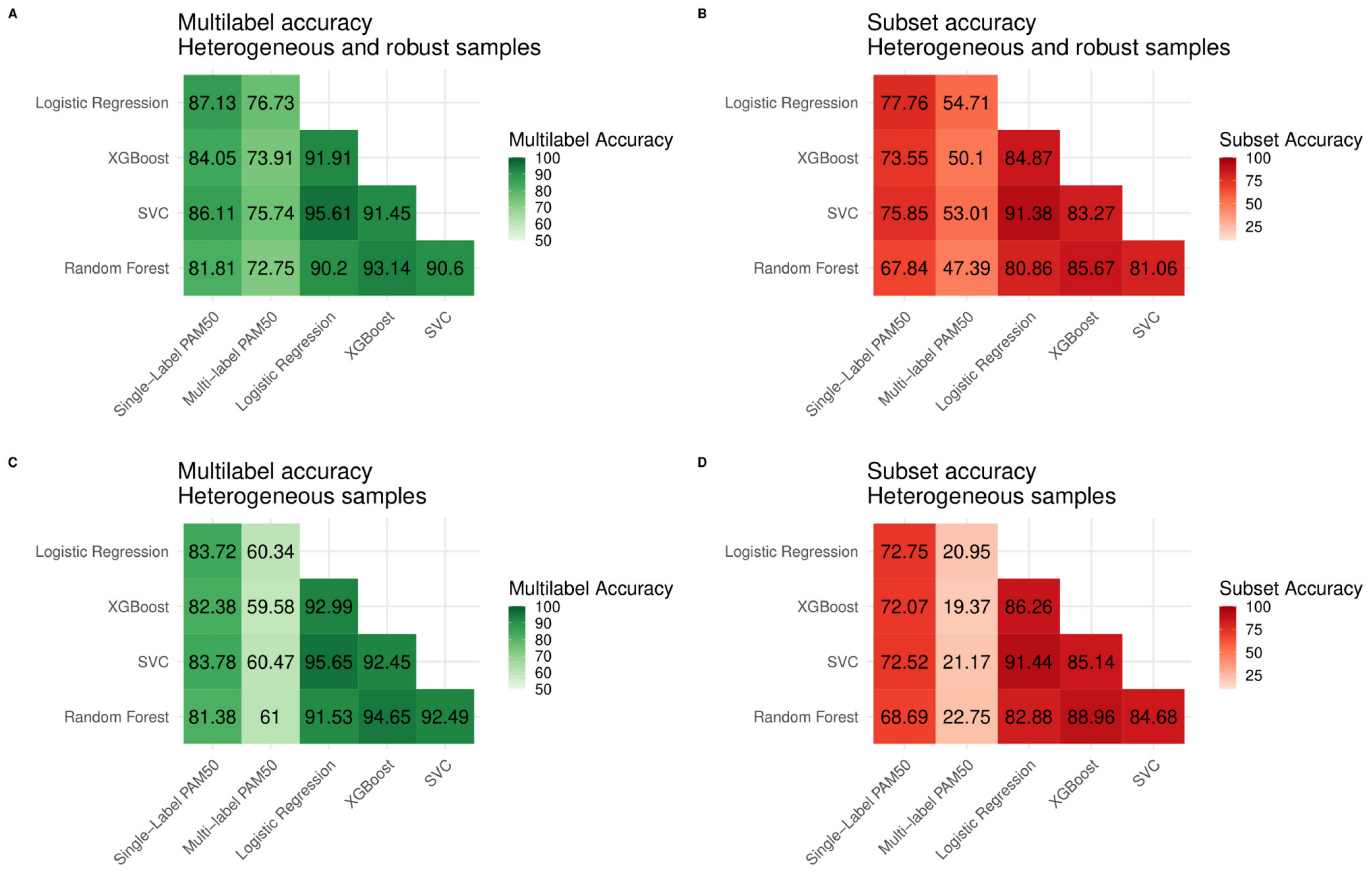
Metrics for robust and heterogeneous samples. Multilabel accuracy **(A)** and subset accuracy **(B)** for heterogeneous and robust samples. Multilabel accuracy **(C)** and subset accuracy **(D)** only for heterogeneous samples. Comparison of multi-label accuracy and subset accuracy for heterogeneous samples mixed with robust samples and heterogeneous samples alone. The metrics are calculated between all algorithms, the single reference PAM50 label and the multi-label PAM50 reference.

Heterogeneous samples were therefore analyzed separately as well, in order to assess whether their complex subtype assignments could be consistently reproduced across classifiers and to further investigate the issue raised by the Normal-like-related class. A direct comparison of heterogeneous samples multi-label characterizations identified through PAM50-based conformal prediction and the prediction sets obtained from nested conformal prediction combined with machine learning predictors is reported in Supplementary Table S3. In particular, the discrepancies observed in subset accuracy with respect to the PAM50 multi-label assignments appear to be driven largely by Normal-like-related mixed prediction sets. Nevertheless, the classifiers seemed highly concordant with each other, suggesting that the disagreement may not reflect random instability but rather a systematic difference in how model-based predictors and PAM50-based multi-label characterizations represent subtype ambiguity. This further motivated a dedicated cross-model comparison of multi-label assignments derived with NCPA. Multi-label and subset accuracy were therefore calculated for each model type against every other model, both for heterogeneous samples alone and for heterogeneous and robust samples together. These results are summarized in Figure 5, together with comparisons against the PAM50 single-label and multi-label classifications. Multi-label accuracies showed high levels of concordance among all classifiers when compared to one another, but consistently lower agreement with the multi-label assignments provided by the PAM50-based conformal framework. This lower agreement may partly reflect the larger average size of PAM50-derived prediction sets, consistent with the higher specificity observed for all machine learning based classifiers. The more stringent subset accuracy analysis showed a similar pattern: high agreement among classifiers, but lower similarity with both the PAM50 single-label and PAM50 multi-label classifications. The detailed examination of prediction sets in Supplementary Table S3 highlights a particularly strong concordance among classifiers in not reproducing the numerous mixed Normal-like/Luminal A and Normal-like/Basal prediction sets emerging from the PAM50-based conformal analysis. This result, also reflected in the concordance patterns shown in Figure 5, reinforces the idea that the PAM50-based conformal prediction may be strongly affected by the problematic contribution of the Normal-like class. Importantly, the high pairwise concordance among classifiers in terms of multi-label and subset accuracy supports the hypothesis that at least a subset of these samples is truly heterogeneous in nature, not simply difficult to classify. In fact, the models appear to converge on a shared notion of subtype complexity, even when this does not perfectly match the prediction sets produced by the PAM50-based conformal procedure.

### 4.4 Weak samples

In this section, we investigate *weak* samples as defined by the conformal analysis performed on PAM50. Weak samples were not assigned to any class in the PAM50-based multi-label setting, and were therefore analyzed according to their original PAM50 single-label subtype to investigate whether this lack of assignment reflected PAM50-based multi-label classification uncertainty or intrinsic sample ambiguity. As reported in Table 2, the HER2-enriched class displayed the highest percentage of samples falling into the weak category (20%), whereas, in all other classes, weak samples are well below 10%, with Basal showing the lowest percentage (1.60%).

In branch B, for each classification algorithm, weak samples were analyzed by comparing their multi-label assignments (derived from conformal prediction sets) with the original PAM50 single-label assignment. As summarized in Supplementary Table S4, multi-label predictions were divided into *exact* when matching the original PAM50 single label, *partial* when the PAM50 single label was included within the prediction set together with additional labels, and *wrong* when the PAM50 label was absent from the prediction set. In addition, the proportion of *complex* predictions, i.e., prediction sets containing more than two labels, was also quantified. Among the weak PAM50 Basal tumors, two out of three were consistently classified as heterogeneous Basal/HER2, only partially confirming the PAM50 original label. Conversely, the third one was classified differently by all methods: LR classified it as LumA/Normal-like, while the other classifiers all predicted large prediction set (three labels by SVC and RF, all five labels by XGB), with LumA being the common label among all of them. Given the generally strong and distinctive characterization of Basal tumors, also confirmed in this work, it is not surprising that the few samples classified as weak in the PAM50-based conformal analysis were also hard to classify by independent trained predictors. In this case, weak samples appear to represent genuinely ambiguous cases affected by prediction uncertainty, rather than limitations or errors in model classification.

Weak samples originally assigned to the single-label PAM50 Normal-like class showed the highest proportions of complex prediction sets across all predictors. This result is coherent with the observations made in the previous analyses, where Normal-like samples were already associated with extensive ambiguity, both in robust and heterogeneous categories. The tendency of weak Normal-like samples to generate broad or unstable prediction sets further supports the interpretation of this class as particularly controversial and difficult to define in a consistent and biologically/clinically relevant way. For weak samples originally assigned to the HER2-enriched PAM50 class, it is worth noting the high proportions of wrong or partial predictions, which once again highlight the intrinsic difficulty in clearly recognizing this subtype. In this case, uncertainty does not appear to be limited to occasional borderline samples, but may instead reflect a more general lack of sharp separation between HER2-enriched tumors and neighboring molecular subtypes, particularly Luminal B. Weak samples originally assigned to the PAM50 Luminal A and Luminal B classes showed high proportions of partial predictions and discrete amounts of exact predictions. However, their prevalence within the weak category remained limited (3.19% and 8.92%, respectively). Therefore, the results of the machine learning predictors simply reinforce the notion that these classes are overall well-distinguishable from the other subtypes, while showing only a biologically reasonable amount of overlap between each other (being both luminal tumors), already observed also in the Heterogeneous category.

Overall, multi-label predictions of the different machine learning models combined with NCPA showed substantial agreement. In particular, complex prediction sets were disproportionately concentrated within this weak category, suggesting that a large fraction of highly multi-label or poorly resolved predictions emerging from the classifiers involve samples that were already not confidently assignable for the PAM50-based conformal analysis. Nonetheless, in Branch B, a subset of weak samples was repeatedly assigned to coherent two-label prediction sets, including the original PAM50 single label together with an additional biologically relevant subtype. This suggests that the proposed approach combining machine learning models and nested conformal prediction analysis may provide a more informative and uncertainty-aware representation of complex cases in which the PAM50-based multi-label assignment fails to produce a usable classification. Furthermore, this reliability could be further enhanced by adopting a consensus strategy across our multiple predictors combined with NCPA.

## 5 Discussion

In this work, we showed that conformal prediction can be systematically integrated into a computational framework to extend the information typically obtained from standard single-label classifiers. More specifically, the proposed approach provides a statistically grounded way to move from a single-label perspective of cancer subtyping toward a multi-label one, in which prediction uncertainty becomes part of the biological and methodological interpretation. Importantly, the framework proved effective even when applied to a relatively small and unbalanced dataset, supporting its practical value in realistic omics settings, where large and balanced cohorts are often unavailable.

A first key aspect of this work is that in Branch A, we apply conformal prediction directly to the state-of-the-art classifier, here the PAM50 method (not requiring any training) to generate more informative multi-label sample characterizations while also revealing subtype-specific patterns of prediction complexity. This allowed samples to be stratified into *robust, heterogeneous*, and *weak* categories, highlighting that in breast cancer subtypes ambiguity and heterogeneity are not uniformly distributed across classes.

Subtype-specific patterns were also biologically coherent. Basal tumors emerged as the most robustly characterized class, consistent with their marked transcriptional identity, whereas Luminal A and Luminal B showed structured overlap, reflecting their expected biological similarity. HER2-enriched tumors displayed a higher proportion of weak and heterogeneous assignments, confirming the greater difficulty in clearly delimiting this subtype. Finally, the Normal-like class showed the highest degree of ambiguity across analyses, supporting the hypothesis that this class may derive from classification-system uncertainty rather than from a consistently separable biological subtype.

Branch B instead offers a complementary analysis by testing whether the multi-label characterizations emerged from Branch A could be traced across independently trained machine-learning predictors combined with our strategy of NCPA. The results confirm that the transition from single-label to multi-label classification captures a non-negligible fraction of true subtype complexity that is consistently recognized across models. In particular, a subset of heterogeneous samples was repeatedly assigned to the same or highly similar prediction sets by different classifiers, indicating that at least part of the observed ambiguity is not randomly due to the model but reflects within-sample complexity. In addition, Branch B showed that not all ambiguity observed in Branch A is reproducible: some broader prediction sets, especially several Normal-like-related mixed assignments, were not consistently supported by the independent classifiers and some weak assignments are solved uniformly using machine learning predictors. This indicates that the proposed two-branch framework is useful not only for detecting heterogeneity, but also for distinguishing sample-derived uncertainty from uncertainty derived from instability/errors in the classification system or method.

The separate analysis of *robust, heterogeneous*, and *weak* samples further supports this interpretation. Robust samples were generally recognized and, to a good extent, maintained single-label by the classifiers, confirming that the framework does not tend to inflate uncertainty when the molecular signal is strong. Heterogeneous samples represented the most informative category for the transition to a multi-label setting, as they can demonstrate the limitations of enforcing single-label subtyping. Weak samples, finally, proved particularly informative rather than merely problematic: although not assigned to any class in the PAM50-based multi-label characterization, a subset of them was repeatedly mapped by Branch B to coherent two-label prediction sets, including the original PAM50 subtype together with another class. This suggests that some apparently unresolved cases may still contain multi-label information.

An additional relevant finding is that the different machine-learning predictors used in Branch B showed substantial agreement with one another, in some cases greater than with the original PAM50-based multi-label assignments. Although this should not be interpreted as evidence that these classifiers are more correct, it does indicate that part of the uncertainty in the PAM50-based multi-label characterizations, particularly for weak samples, may solved with other feature spaces and algorithms. This also reinforces the need for an uncertainty-aware interpretation of PAM50 subtype labels, especially considering that these are used as a supervised reference for other predictive models. Hence, a promising future direction could be the adoption of a consensus strategy across multiple predictors combined with NCPA.

From a methodological perspective, one of the main advantages of the proposed framework is its suitability for small and unbalanced datasets. Conformal prediction does not require strong distributional assumptions and can be flexibly combined with different underlying classifiers. Nonetheless, so far was only used in extremely high data context. Our innovative NCPA makes it adequate for omics applications, where sample sizes are often limited and uncertainty quantification is essential. Overall, these findings indicate that prediction sets allow intrinsic tumor ambiguity to emerge at the level of individual samples, enabling a more critical and biologically informed interpretation of subtype assignments. Rather than treating all predictions as equally reliable, the proposed framework makes it possible to distinguish between well-supported classifications, structured heterogeneity, and genuinely unresolved cases, thereby providing a principled bridge between uncertainty quantification and the biological complexity of cancer subtyping.

## 6 Conclusions

The central contribution of this work lies in the design of an innovative approach and its implementation in a computational framework that employs a novel strategy of conformal prediction to enable a rigorous transition from multi-class to multi-label classification in cancer subtyping. Our approach addresses the challenge posed by intra-sample heterogeneity and overcomes key limitations of traditional subtyping approaches, based on multi-class classification strategies that enforce mutually exclusive subtype assignments. By leveraging conformal prediction and integrating it in our analytical framework, we proposed a shift toward a multi-label perspective, in which prediction uncertainty is explicitly modeled and interpreted. Furthermore, the developed methodology makes the approach particularly valuable for small and unbalanced sample settings, typical of omics studies, where uncertainty estimation is critical and standard outputs may be unreliable. Indeed, our framework integrates multi-stage data partitioning within exhaustive combinatorial fold structures, generating a stochastic distribution of conformal thresholds able to mitigate the variance issue due to data scarcity and heterogeneity. The complementary and comparative evaluations of Branch A and B represent one of the main strengths of the present work: while Branch A identifies possible uncertainty in the state-of-the-art subtyping method and classification, Branch B evaluates whether derived multi-label characterization are robust enough to persist across machine learning models and data perturbations. Our results demonstrate that our new nested conformal prediction strategy provides a simple yet effective way to quantify and interpret uncertainty in subtype classification.

A further contribution of this work is the categorization of different forms of prediction complexity. Across both experimental branches—using reference PAM50 labels in Branch A and predictions from multiple machine-learning models in Branch B—we observed not only robust single-label predictions, but also consistent evidence of heterogeneous patterns as well as different problem types underneath “weak” assignments. By identifying samples that are consistently associated with multiple labels across models and runs, we highlighted the clear need for multi-label prediction to better describe tumor inner intricacy. These findings, in fact, reinforce the notion that single-label predictions may be insufficient to capture the full intrinsic molecular variability of cancer samples. Furthermore, given the substantial agreement observed among classifiers, a consensus-based aggregation of prediction sets could further enhance robustness, reduce model-specific instability, and improve the reliability of multi-label subtype assignments, especially in hard-to-predict cases. Additionally, the consensus based aggregation of trained models predictions and corresponding conformal thresholds, can be used to provide a reliable multi-label classification for any new single sample, used as an independent test sample.

Although the proposed framework allows uncertainty in class prediction to be quantified and classification tasks to be extended to a multi-label setting, several limitations should be acknowledged. First, the approach relies on pre-existing classification systems and state-of-the-art classifiers, which may themselves carry assumptions, biases, or limitations in representing the full biological complexity of the data. Second, this work was conducted on a single breast cancer transcriptomic dataset and focused on one established subtyping method, the PAM50. Therefore, further validation on independent datasets, varying in size and class proportions, and on additional cancer types, will be necessary to assess the reproducibility and generalizability of the framework, especially given its statistical and exploratory nature. In addition, the methodology uses PAM50 labels both in Branch A and B; although PAM50 is a well-established, state-of-the-art approach in breast cancer subtype classifications, the conclusions drawn related to the comparisons of the two branches could be susceptible to this single methodology of sample labeling. To further evaluate the biological relevance of the findings obtained here, future analyses should investigate whether the newly identified multi-label associations correspond to differences in disease progression, prognosis, treatment response, or other clinically relevant outcomes. This would help clarify the biological and clinical significance of the identified predictions and could ultimately support a translational use of the framework. However, our results suggest that prediction sets can provide a more informative representation of tumor complexity than single-label assignments alone, even in borderline cases, by leveraging machine learning predictors and consensus strategies.

Overall, this work broadens the application of conformal prediction and supports its use as a robust methodology for uncertainty-aware cancer subtyping, also in small and unbalanced data contexts. By providing a flexible yet statistically grounded representation of tumor heterogeneity, our approach may contribute to a more detailed and realistic interpretation of molecular classification not only in breast or other cancer subtyping, but potentially across a wider range of complex disease scenarios.

## Data Availability

Publicly available datasets were analyzed in this study. This data can be found here: https://portal.gdc.cancer.gov/projects/TCGA-BRCA.
