# Supplementary material for: Novel nested conformal prediction analysis to unravel complexity in patient subtyping
Source: Front Artif Intell. 2026 Jul 20;9:1844254. doi: 10.3389/frai.2026.1844254 (PMC13429844; doi:10.3389/frai.2026.1844254)
Supplement: Supplementary file 1 [file Data_Sheet_1.pdf]

# Supplementary Material

## 1 SUPPLEMENTARY TABLES AND FIGURES

### 1.1 Tables

| PAM50 Single-Label | PAM50-based Conformal Prediction Sets<br>Multi-label | Count | Frequency |
|---|---|---|---|
| **LumA** (565) | LumA | 249 | 44.07% |
| | LumA, Normal-like | 268 | 47.43% |
| | LumA, LumB | 29 | 5.13% |
| | LumA, HER2 | 1 | 0.18% |
| | Empty | 18 | 3.19% |
| **Basal** (188) | Basal | 145 | 77.13% |
| | Basal, HER2 | 2 | 1.06% |
| | Basal, Normal-like | 38 | 20.21% |
| | Empty | 3 | 1.60% |
| **LumB** (157) | LumB | 101 | 64.33% |
| | LumB, LumA | 33 | 21.02% |
| | LumB, HER2 | 9 | 5.73% |
| | Empty | 14 | 8.92% |
| **HER2** (72) | HER2 | 47 | 65.28% |
| | HER2, LumB | 8 | 11.11% |
| | Empty | 15 | 20.83% |
| **Normal-like** (71) | Normal-like | 12 | 16.90% |
| | Normal-like, LumA | 49 | 69.01% |
| | Normal-like, Basal | 5 | 7.04% |
| | Empty | 5 | 7.04% |

Table S1: **PAM50 conformal prediction sets with respect to PAM50 label** PAM50 ’ground truth’ single label assignment is compared to its corresponding new multi-label assignments obtained through conformal prediction application.

| **PAM50 Single-Label:** | | **LumA** | **LumB** | **HER2** | **Basal** | **Normal-like** |
|---|---|---|---|---|---|---|
| | **Total** | 249 | 101 | 47 | 145 | 12 |
| **Logistic Regression** | Exact | 85.14% | 60.4% | 82.98% | 95.86% | 16.67% |
| | Partial | 14.46% | 37.62% | 12.77% | 3.45% | 75.00% |
| | Wrong | 0.40% | 1.98% | 4.26% | 0.69% | 8.33% |
| **XGB** | Exact | 83.94% | 46.53% | 40.43% | 95.86% | 0.00% |
| | Partial | 16.06% | 50.50% | 55.32% | 3.45% | 75.00% |
| | Wrong | 0.00% | 2.97% | 4.26% | 0.69% | 25.00% |
| **SVC** | Exact | 81.53% | 55.45% | 78.72% | 95.17% | 8.33% |
| | Partial | 18.07% | 39.60% | 17.02% | 4.14% | 83.33% |
| | Wrong | 0.40% | 4.95% | 4.26% | 0.69% | 8.33% |
| **Random Forest** | Exact | 74.70% | 30.69% | 36.17% | 95.17% | 0.00% |
| | Partial | 25.3% | 67.33% | 57.45% | 4.14% | 58.33% |
| | Wrong | 0.00% | 1.98% | 6.38% | 0.69% | 41.67% |

Table S2: **Robust samples classification**. Robust samples distribution of exact, wrong and partial predictions according to their PAM50 assignments

| **Class** | **Pam50 Count** | **Logistic Regression** | **XGB** | **SVC** | **RF** |
|---|---|---|---|---|---|
| LumA | 0 | 257 (0%) | 267 (0%) | 258 (0%) | 260 (0%) |
| Basal | 0 | 36 (0%) | 36 (0%) | 37 (0%) | 37 (0%) |
| LumB | 0 | 13 (0%) | 7 (0%) | 8 (0%) | 5 (0%) |
| HER2 | 0 | 3 (0%) | 2 (0%) | 4 (0%) | 0 (0%) |
| Normal-like | 0 | 14 (0%) | 8 (0%) | 15 (0%) | 3 (0%) |
| LumA, HER2 | 1 | 0 (0%) | 0 (0%) | 0 (0%) | 0 (0%) |
| LumA, LumB | 62 | 32 (51.61%) | 36 (58.06%) | 37 (59.68%) | 40 (64.52%) |
| LumA, Normal-like | 317 | 49 (15.46%) | 37 (11.67%) | 46 (14.51%) | 49 (15.46%) |
| LumB, HER2 | 17 | 4 (23.53%) | 3 (17.65%) | 4 (23.53%) | 3 (17.65%) |
| HER2, Basal | 4 | 2 (50.00%) | 3 (75.00%) | 3 (75.00%) | 4 (100%) |
| Basal, Normal-like | 43 | 6 (13.95%) | 7 (16.28%) | 4 (9.30%) | 5 (11.63%) |

Table S3: **Heterogeneous samples classification**. Heterogeneous samples classifications obtained multi-label predictions compared against their PAM50 multi-label classification.

| PAM50 Single-Label: | | LumA | LumB | HER2 | Normal-like | Basal |
|---|---|---|---|---|---|---|
| | **Total** | 18 | 14 | 15 | 5 | 3 |
| **LR** | Exact | 3 | 5 | 6 | 1 | 1 |
| | Partial | 12 | 9 | 7 | 3 | 1 |
| | Wrong | 3 | 0 | 2 | 1 | 1 |
| | Complex | 22.22% | 7.14% | 46.67% | 0.00% | 0.00% |
| **XGB** | Exact | 4 | 1 | 1 | 0 | 0 |
| | Partial | 13 | 13 | 14 | 4 | 3 |
| | Wrong | 1 | 0 | 0 | 1 | 0 |
| | Complex | 27.78% | 21.43% | 86.67% | 60.00% | 33.33% |
| **SVM** | Exact | 1 | 2 | 5 | 0 | 0 |
| | Partial | 16 | 12 | 6 | 4 | 2 |
| | Wrong | 1 | 0 | 4 | 1 | 1 |
| | Complex | 16.67% | 14.29% | 33.33% | 60.00% | 33.33% |
| **RF** | Exact | 3 | 1 | 2 | 0 | 0 |
| | Partial | 15 | 13 | 10 | 3 | 2 |
| | Wrong | 0 | 0 | 3 | 2 | 1 |
| | Complex | 16.67% | 0.00% | 33.33% | 40.00% | 0.00% |

Table S4: **Weak samples classification**. Weak samples count of exact, wrong and partial multi-label predictions according to their PAM50 original single-label assignments.

## 1.2 Figures

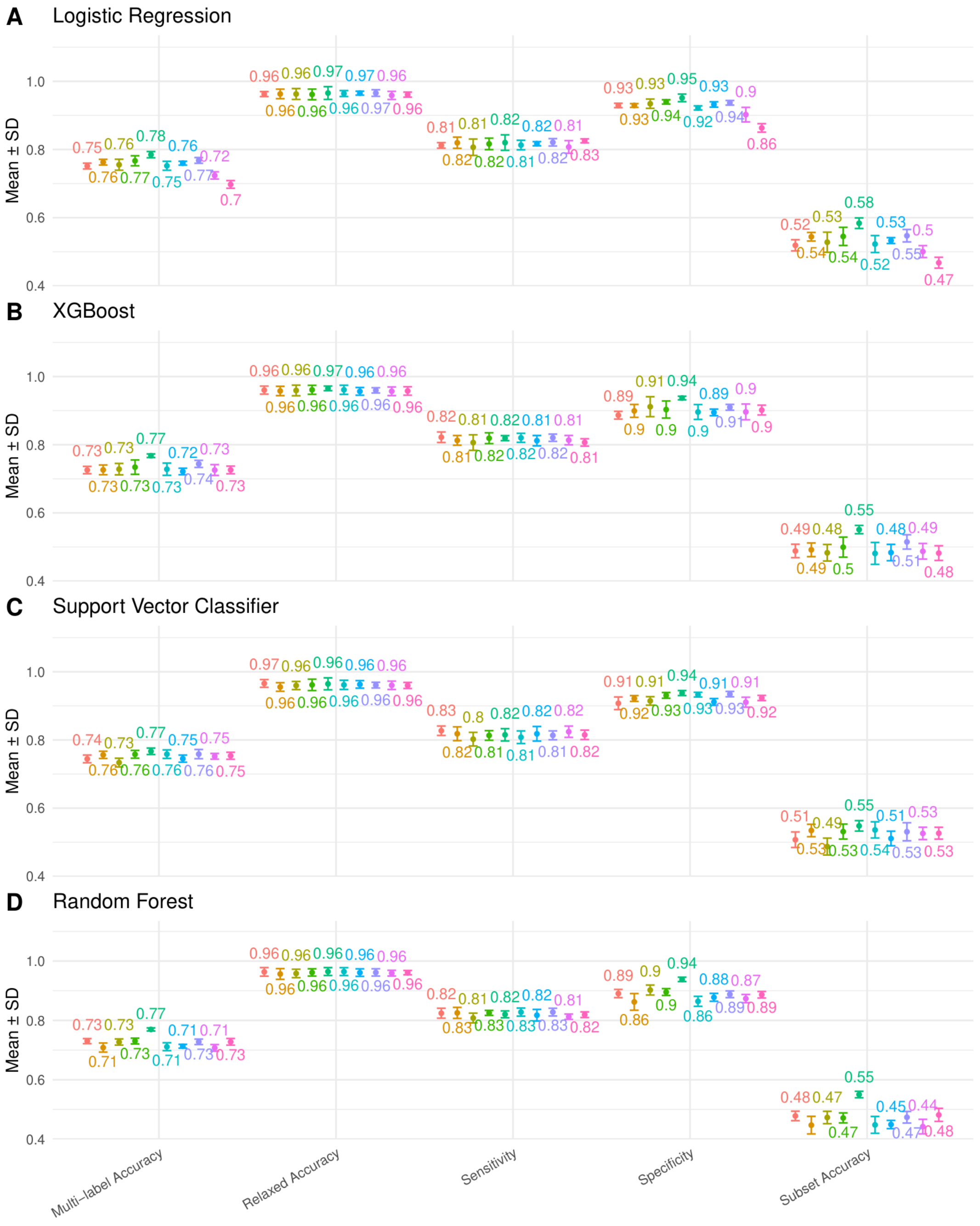


Figure S1: **Multi-label metrics for heterogeneous and robust samples**. Metrics described with mean and standard deviation for each of the 10 machine learning runs. The metrics are calculated independently for each conformal prediction run performed in the 10 runs. The reference label considered is the multi-label PAM50 assignment.
